# (3a*R*,6*S*,7a*R*)-7a-Bromo-2-methyl­sulfonyl-1,2,3,6,7,7a-hexa­hydro-3a,6-ep­oxy­isoindole

**DOI:** 10.1107/S1600536811015959

**Published:** 2011-05-07

**Authors:** Ersin Temel, Aydın Demircan, Hakan Arslan, Orhan Büyükgüngör

**Affiliations:** a Department of Physics, Arts and Sciences Faculty, Ondokuz Mayıs University, Samsun, TR 55139, Turkey; b Department of Chemistry, Faculty of Arts and Sciences, Nigde University, Nigde, TR 51240, Turkey; c Department of Chemistry, Emory University, Atlanta, GA 30322, USA; d Department of Chemistry, Faculty of Arts and Science, Mersin University, Mersin, TR 33343, Turkey

## Abstract

In the title compound, C_9_H_12_BrNO_3_S, the two tetra­hydro­furan rings adopt envelope conformations, the pyrrolidine ring adopts a half-chair conformation and the six-membered ring is in a boat conformation. In the crystal, weak inter­molecular C—H⋯O hydrogen bonds link the mol­ecules into *R* ^2^_2_(8) and *R* ^2^_2_(14) rings along the *b-*axis direction.

## Related literature

For a related structure, see: Koşar *et al.* (2006 ▶). For uses of sulfonamides in medicine, in particular the treatment of bacterial infection, see: Kleemann *et al.* (1999 ▶); Cremlyn (1996 ▶). For the synthesis of sulfonamides, see: Anderson (1979 ▶). For thermal intra­molecular Diels–Alder reaction of furans (IMDAF), see: Demircan & Parsons (2002 ▶); Arslan *et al.* (2008 ▶). A mesyl group in the structure is normally chosen as a protective group for nitro­gen, but at the same time accelerates the cyclo­addition process for IMDAF, see: Greene (1981 ▶); Choony *et al.* (1997 ▶). For standard bond lengths, see: Allen *et al.* (1987 ▶). For puckering parameters, see: Cremer & Pople (1975 ▶). For graph-set notation, see: Bernstein *et al.* (1995 ▶). 
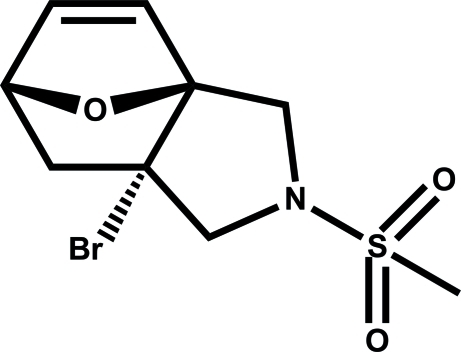


## Experimental

### 

#### Crystal data

C_9_H_12_BrNO_3_S
*M* *_r_* = 294.17
Triclinic, 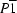

*a* = 5.9478 (7) Å
*b* = 9.5869 (10) Å
*c* = 10.7775 (11) Å
α = 114.307 (8)°
β = 90.481 (9)°
γ = 97.812 (9)°
*V* = 553.46 (10) Å^3^
*Z* = 2
Mo *K*α radiation
μ = 3.89 mm^−1^
*T* = 296 K
0.38 × 0.23 × 0.08 mm

#### Data collection

Stoe IPDS 2 diffractometer
Absorption correction: integration (*X-RED32*; Stoe & Cie, 2001 ▶) *T*_min_ = 0.223, *T*_max_ = 0.682
8163 measured reflections
2293 independent reflections
1920 reflections with *I* > 2σ(*I*)
*R*_int_ = 0.082

#### Refinement

*R*[*F* ^2^ > 2σ(*F* ^2^)] = 0.047
*wR*(*F* ^2^) = 0.107
*S* = 1.10
2293 reflections
137 parameters
H-atom parameters constrained
Δρ_max_ = 0.86 e Å^−3^
Δρ_min_ = −0.44 e Å^−3^

### 

Data collection: *X-AREA* (Stoe & Cie, 2002 ▶); cell refinement: *X-AREA*; data reduction: *X-RED32* (Stoe & Cie, 2001 ▶); program(s) used to solve structure: *SHELXS97* (Sheldrick, 2008 ▶); program(s) used to refine structure: *SHELXL97* (Sheldrick, 2008 ▶); molecular graphics: *OLEX2 *(Dolomanov *et al.*, 2009 ▶); software used to prepare material for publication: *SHELXTL* (Sheldrick, 2008 ▶), *OLEX2*, *publCIF* (Westrip, 2010 ▶) and *Mercury* (Macrae *et al.*, 2006 ▶).

## Supplementary Material

Crystal structure: contains datablocks I, global. DOI: 10.1107/S1600536811015959/hg5029sup1.cif

Structure factors: contains datablocks I. DOI: 10.1107/S1600536811015959/hg5029Isup2.hkl

Supplementary material file. DOI: 10.1107/S1600536811015959/hg5029Isup3.cml

Additional supplementary materials:  crystallographic information; 3D view; checkCIF report

## Figures and Tables

**Table 1:** Hydrogen-bond geometry (Å, °)

*D*—H⋯*A*	*D*—H	H⋯*A*	*D*⋯*A*	*D*—H⋯*A*
C9—H9*A*⋯O2^i^	0.96	2.59	3.385 (7)	140
C9—H9*C*⋯O1^ii^	0.96	2.59	3.540 (6)	172

Symmetry codes: (i) 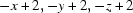; (ii) 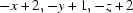.

**Fig. 1.**
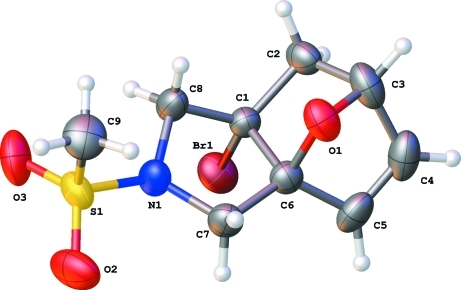
The molecular structure of the title compound, with displacement ellipsoids drawn at the 30% probability level.

**Fig. 2.**
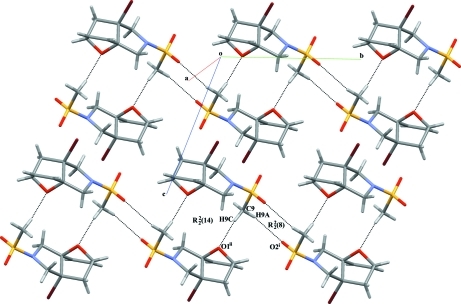
Part of the crystal structure of the title compound, showing the formation of R22(8) and R22(14) rings along [010]. Hydrogen bonds are indicated by dashed lines. (Symmetry codes as in Table 1)

**Fig. 3.**
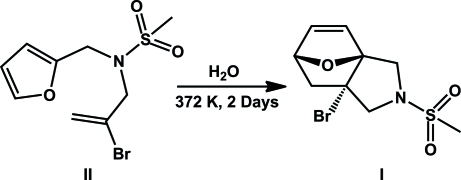
Synthesis of the title compound.

## supplementary crystallographic information

### Comment

Sulfonamides are one of the most important groups of compounds for the medical purposes (Kleemann *et al.*, 1999). They have mostly been applied for the treatment of bacterial infection (Cremlyn, 1996). Sulfonamide is not only an early class of antibiotics, also, has showed different functionality such as a protease inhibitor amprenavir, the analgesic celecoxib, sildenafil for erectile dysfunction, and the antimigraine agent sumatriptan. The most sulfonamides have been synthesized from a reaction between a sulfonyl chloride and ammonia or primary or secondary amines or *via* related transformations (Anderson, 1979).

Several thermal intramolecular Diels Alder reaction of furans (IMDAF) cycloadditon were performed including a nitrogen linked side chain of furan and already reported by Demircan and his co-workers (Demircan and Parsons, 2002; Arslan *et al.*, 2008). We would like to report here a newly synthesized sulfonamide, I.

In continuation of our interest, mesyl group in the structure is normally chosen as a protective group for nitrogen, but at the same time, accelerates the cycloaddition process for IMDAF (Greene, 1981; Choony *et al.*, 1997). This facile, versatile and environmentally friendly reaction was accomplished in aqueous condition and stirred for two days at 372 K.

The molecular structure of the title compound, I, is shown in Fig. 1. A l l bond lengths show normal values (Allen *et al.*, 1987). In addition, the C—Br bond distance, 1.953 (4) Å, is not significantly different from the value reported for C—Br single bond (1.961 (3) Å; Koşar *et al.*, 2006). The six membered ring, C1—C6, is in a boat conformation with puckering parameter *Q* = 0.933 (5) Å, *θ* = 89.1 (3) °, *φ* = 119.1 (3) °. The tetrahydrofuran, O1/C3–C6, and bromo-attached tetrahydrofuran, O1/C3/C2/C1/C6, rings adopt envelope conformations, and the puckering parameter *Q* values are 0.517 (5) and 0.607 (4) ° Å, respectively (Cremer & Pople, 1975). The pyrrolidine ring, N1/C7/C6/C1/C8, adopts half chair conformation, and the puckering parameter *Q* = 0.368 (4) Å and *φ* = 304.8 (7) °, (Cremer & Pople, 1975).

Fig. 2 shows the packing of the molecules in the unit cell. The crystal packing of (I) is stabilized by intermolecular C9—H9A···O2 and C9—H9C···O1 interactions (Table 1). The methyl group in the reference molecule at (*x*, *y*, *z*) acts as double hydrogen bond donor, *via* H9A and H9C, to atoms O2 in the molecule at (-*x* + 2, -*y* + 2, -*z* + 2) and O1 in the molecule at (-*x* + 2, -*y* + 1, -*z* + 2), so forming successive *R*^2^_2_(8) and *R*^2^_2_(14) rings running parallel to the [010] direction (Bernstein *et al.*, 1995).

### Experimental

*N*-(2-bromoprop-2-en-1-yl)-*N*-(2-furylmethyl)methanesulfonamide, II, (1 g, 3.4 mmol) was stirred in water (25 ml) at 372 K for two days (Fig. 3). The reaction was stirred and monitored by thin layer chromatography until no further cycloadditon observed. The mixture was poured into ethylacetate (25 ml) and aqueous phase was washed with excess of ethyl acetate (2 *x* 25 ml). Combined organic phases was dried on magnesium sulfate and filtered off. The solvent was then removed under reduced pressure. The residue was subjected to flash column chromatography to afford the title compound, (3a*R*,6*S*,7a*R*)-7a-bromo-2-(methylsulfonyl)-1,2,3,6,7,7a-hexahydro-3a,6-epoxyisoindole, I, with 0.74 g, 74% yield as light brown crystals.

### Refinement

H atoms were positioned geometrically and treated using a riding model, fixing the bond lengths at 0.96, 0.97, 0.98 and 0.93 Å for CH_3_, CH_2_, CH and CH(aromatic), respectively. The displacement parameters of the H atoms were constrained with *U*_iso_(H) = 1.2*U*_eq_ (aromatic, methylene or methine C) or 1.5*U*_eq_ (methyl C).

### Crystal data

**Table:**

C_9_H_12_BrNO_3_S	*Z* = 2
*M**_r_* = 294.17	*F*(000) = 296
Triclinic, *P*1	*D*_x_ = 1.765 Mg m^−^^3^
Hall symbol: -P 1	Mo *K*α radiation, λ = 0.71073 Å
*a* = 5.9478 (7) Å	Cell parameters from 8163 reflections
*b* = 9.5869 (10) Å	θ = 2.1–27.5°
*c* = 10.7775 (11) Å	µ = 3.89 mm^−^^1^
α = 114.307 (8)°	*T* = 296 K
β = 90.481 (9)°	Block, light-brown
γ = 97.812 (9)°	0.38 × 0.23 × 0.08 mm
*V* = 553.46 (10) Å^3^	

### Data collection

**Table:**

Stoe IPDS 2 diffractometer	2293 independent reflections
Radiation source: fine-focus sealed tube	1920 reflections with *I* > 2σ(*I*)
graphite	*R*_int_ = 0.082
Detector resolution: 6.67 pixels mm^-1^	θ_max_ = 26.5°, θ_min_ = 2.1°
rotation method scans	*h* = −7→7
Absorption correction: integration (*X-RED*; Stoe & Cie, 2001)	*k* = −12→12
*T*_min_ = 0.223, *T*_max_ = 0.682	*l* = −13→13
8163 measured reflections	

### Refinement

**Table:**

Refinement on *F*^2^	Primary atom site location: structure-invariant direct methods
Least-squares matrix: full	Secondary atom site location: difference Fourier map
*R*[*F*^2^ > 2σ(*F*^2^)] = 0.047	Hydrogen site location: inferred from neighbouring sites
*wR*(*F*^2^) = 0.107	H-atom parameters constrained
*S* = 1.10	*w* = 1/[σ^2^(*F*_o_^2^) + (0.0452*P*)^2^ + 0.3277*P*] where *P* = (*F*_o_^2^ + 2*F*_c_^2^)/3
2293 reflections	(Δ/σ)_max_ < 0.001
137 parameters	Δρ_max_ = 0.86 e Å^−^^3^
0 restraints	Δρ_min_ = −0.44 e Å^−^^3^

### Special details

**Table:**

Geometry. All e.s.d.'s (except the e.s.d. in the dihedral angle between two l.s. planes) are estimated using the full covariance matrix. The cell e.s.d.'s are taken into account individually in the estimation of e.s.d.'s in distances, angles and torsion angles; correlations between e.s.d.'s in cell parameters are only used when they are defined by crystal symmetry. An approximate (isotropic) treatment of cell e.s.d.'s is used for estimating e.s.d.'s involving l.s. planes.
Refinement. Refinement of *F*^2^ against ALL reflections. The weighted *R*-factor *wR* and goodness of fit *S* are based on *F*^2^, conventional *R*-factors *R* are based on *F*, with *F* set to zero for negative *F*^2^. The threshold expression of *F*^2^ > σ(*F*^2^) is used only for calculating *R*-factors(gt) *etc*. and is not relevant to the choice of reflections for refinement. *R*-factors based on *F*^2^ are statistically about twice as large as those based on *F*, and *R*- factors based on ALL data will be even larger.

### Fractional atomic coordinates and isotropic or equivalent isotropic displacement parameters (Å^2^)

**Table:**

	*x*	*y*	*z*	*U*_iso_*/*U*_eq_	
C1	0.7380 (6)	0.3068 (4)	0.6385 (4)	0.0348 (8)	
C2	0.8248 (7)	0.1507 (5)	0.5964 (4)	0.0438 (9)	
H2A	0.9770	0.1543	0.5651	0.053*	
H2B	0.7234	0.0668	0.5264	0.053*	
C3	0.8218 (9)	0.1363 (5)	0.7338 (5)	0.0553 (11)	
H3	0.9189	0.0654	0.7421	0.066*	
C4	0.5780 (9)	0.1066 (6)	0.7645 (5)	0.0577 (12)	
H4	0.4995	0.0132	0.7588	0.069*	
C5	0.4970 (8)	0.2380 (6)	0.8007 (4)	0.0502 (10)	
H5	0.3516	0.2569	0.8268	0.060*	
C6	0.6907 (6)	0.3509 (4)	0.7910 (4)	0.0369 (8)	
C7	0.7123 (7)	0.5249 (5)	0.8604 (4)	0.0441 (9)	
H7A	0.5645	0.5578	0.8801	0.053*	
H7B	0.8083	0.5663	0.9446	0.053*	
C8	0.9051 (7)	0.4468 (4)	0.6497 (4)	0.0429 (9)	
H8A	1.0585	0.4388	0.6744	0.051*	
H8B	0.9039	0.4590	0.5647	0.051*	
O2	0.7873 (6)	0.8440 (4)	0.8963 (4)	0.0738 (11)	
Br1	0.46555 (8)	0.29243 (5)	0.52887 (5)	0.05052 (17)	
N1	0.8186 (6)	0.5743 (4)	0.7581 (3)	0.0443 (8)	
O1	0.8850 (5)	0.2972 (3)	0.8292 (3)	0.0484 (7)	
C9	1.2003 (8)	0.7824 (6)	0.8803 (5)	0.0592 (12)	
H9A	1.2643	0.8899	0.9136	0.089*	
H9B	1.2967	0.7208	0.8158	0.089*	
H9C	1.1881	0.7529	0.9552	0.089*	
O3	0.9632 (6)	0.7717 (4)	0.6764 (4)	0.0633 (9)	
S1	0.92989 (17)	0.75291 (11)	0.80041 (11)	0.0423 (2)	

### Atomic displacement parameters (Å^2^)

**Table:**

	*U*^11^	*U*^22^	*U*^33^	*U*^12^	*U*^13^	*U*^23^
C1	0.0378 (18)	0.034 (2)	0.0349 (19)	0.0078 (15)	0.0081 (15)	0.0151 (15)
C2	0.044 (2)	0.030 (2)	0.053 (2)	0.0081 (16)	0.0068 (18)	0.0124 (17)
C3	0.075 (3)	0.036 (2)	0.059 (3)	0.012 (2)	−0.007 (2)	0.022 (2)
C4	0.083 (3)	0.044 (3)	0.047 (2)	−0.008 (2)	0.002 (2)	0.024 (2)
C5	0.055 (2)	0.055 (3)	0.039 (2)	−0.003 (2)	0.0134 (19)	0.021 (2)
C6	0.0406 (19)	0.038 (2)	0.0328 (19)	0.0069 (15)	0.0043 (15)	0.0146 (16)
C7	0.052 (2)	0.036 (2)	0.041 (2)	0.0093 (17)	0.0121 (17)	0.0124 (17)
C8	0.052 (2)	0.031 (2)	0.043 (2)	0.0058 (17)	0.0165 (18)	0.0135 (17)
O2	0.066 (2)	0.0383 (19)	0.098 (3)	0.0125 (16)	0.012 (2)	0.0082 (18)
Br1	0.0549 (3)	0.0520 (3)	0.0448 (2)	0.01178 (18)	−0.00663 (17)	0.01939 (19)
N1	0.057 (2)	0.0296 (18)	0.0427 (19)	0.0044 (14)	0.0115 (15)	0.0122 (14)
O1	0.0595 (18)	0.0385 (16)	0.0455 (16)	0.0081 (13)	−0.0092 (13)	0.0159 (13)
C9	0.051 (2)	0.067 (3)	0.058 (3)	0.002 (2)	−0.011 (2)	0.027 (3)
O3	0.082 (2)	0.053 (2)	0.067 (2)	−0.0026 (16)	−0.0173 (18)	0.0413 (18)
S1	0.0465 (6)	0.0280 (5)	0.0509 (6)	0.0065 (4)	−0.0034 (4)	0.0148 (4)

### Geometric parameters (Å, °)

**Table:**

C1—C8	1.519 (5)	C6—C7	1.507 (6)
C1—C2	1.538 (5)	C7—N1	1.482 (5)
C1—C6	1.558 (5)	C7—H7A	0.9700
C1—Br1	1.953 (4)	C7—H7B	0.9700
C2—C3	1.542 (6)	C8—N1	1.460 (5)
C2—H2A	0.9700	C8—H8A	0.9700
C2—H2B	0.9700	C8—H8B	0.9700
C3—O1	1.452 (5)	O2—S1	1.422 (4)
C3—C4	1.505 (7)	N1—S1	1.619 (3)
C3—H3	0.9800	C9—S1	1.749 (5)
C4—C5	1.317 (7)	C9—H9A	0.9600
C4—H4	0.9300	C9—H9B	0.9600
C5—C6	1.506 (6)	C9—H9C	0.9600
C5—H5	0.9300	O3—S1	1.432 (3)
C6—O1	1.448 (5)		
			
C8—C1—C2	118.5 (3)	C7—C6—C1	107.2 (3)
C8—C1—C6	101.3 (3)	N1—C7—C6	103.0 (3)
C2—C1—C6	102.7 (3)	N1—C7—H7A	111.2
C8—C1—Br1	108.7 (3)	C6—C7—H7A	111.2
C2—C1—Br1	112.9 (3)	N1—C7—H7B	111.2
C6—C1—Br1	112.0 (2)	C6—C7—H7B	111.2
C1—C2—C3	100.0 (3)	H7A—C7—H7B	109.1
C1—C2—H2A	111.8	N1—C8—C1	102.6 (3)
C3—C2—H2A	111.8	N1—C8—H8A	111.2
C1—C2—H2B	111.8	C1—C8—H8A	111.2
C3—C2—H2B	111.8	N1—C8—H8B	111.2
H2A—C2—H2B	109.5	C1—C8—H8B	111.2
O1—C3—C4	100.7 (4)	H8A—C8—H8B	109.2
O1—C3—C2	101.0 (3)	C8—N1—C7	111.4 (3)
C4—C3—C2	108.5 (4)	C8—N1—S1	121.6 (3)
O1—C3—H3	115.0	C7—N1—S1	120.8 (3)
C4—C3—H3	115.0	C6—O1—C3	95.6 (3)
C2—C3—H3	115.0	S1—C9—H9A	109.5
C5—C4—C3	107.0 (4)	S1—C9—H9B	109.5
C5—C4—H4	126.5	H9A—C9—H9B	109.5
C3—C4—H4	126.5	S1—C9—H9C	109.5
C4—C5—C6	105.1 (4)	H9A—C9—H9C	109.5
C4—C5—H5	127.4	H9B—C9—H9C	109.5
C6—C5—H5	127.4	O2—S1—O3	119.5 (2)
O1—C6—C5	101.4 (3)	O2—S1—N1	105.8 (2)
O1—C6—C7	111.1 (3)	O3—S1—N1	107.14 (19)
C5—C6—C7	125.8 (4)	O2—S1—C9	109.3 (3)
O1—C6—C1	97.9 (3)	O3—S1—C9	106.7 (2)
C5—C6—C1	109.8 (3)	N1—S1—C9	107.9 (2)
			
C8—C1—C2—C3	108.2 (4)	C5—C6—C7—N1	−143.5 (4)
C6—C1—C2—C3	−2.3 (4)	C1—C6—C7—N1	−12.1 (4)
Br1—C1—C2—C3	−123.1 (3)	C2—C1—C8—N1	−148.4 (4)
C1—C2—C3—O1	−35.0 (4)	C6—C1—C8—N1	−37.1 (4)
C1—C2—C3—C4	70.3 (4)	Br1—C1—C8—N1	80.9 (3)
O1—C3—C4—C5	31.9 (5)	C1—C8—N1—C7	32.5 (4)
C2—C3—C4—C5	−73.6 (5)	C1—C8—N1—S1	−175.0 (3)
C3—C4—C5—C6	0.7 (5)	C6—C7—N1—C8	−12.6 (5)
C4—C5—C6—O1	−33.4 (4)	C6—C7—N1—S1	−165.3 (3)
C4—C5—C6—C7	−160.2 (4)	C5—C6—O1—C3	51.1 (4)
C4—C5—C6—C1	69.4 (4)	C7—C6—O1—C3	−173.0 (3)
C8—C1—C6—O1	−84.1 (3)	C1—C6—O1—C3	−61.0 (3)
C2—C1—C6—O1	38.8 (3)	C4—C3—O1—C6	−50.1 (4)
Br1—C1—C6—O1	160.2 (2)	C2—C3—O1—C6	61.4 (4)
C8—C1—C6—C5	170.7 (3)	C8—N1—S1—O2	173.3 (4)
C2—C1—C6—C5	−66.4 (4)	C7—N1—S1—O2	−36.8 (4)
Br1—C1—C6—C5	55.0 (4)	C8—N1—S1—O3	44.7 (4)
C8—C1—C6—C7	31.0 (4)	C7—N1—S1—O3	−165.3 (3)
C2—C1—C6—C7	153.9 (3)	C8—N1—S1—C9	−69.8 (4)
Br1—C1—C6—C7	−84.7 (3)	C7—N1—S1—C9	80.1 (4)
O1—C6—C7—N1	93.8 (4)		

### Hydrogen-bond geometry (Å, °)

**Table:**

*D*—H···*A*	*D*—H	H···*A*	*D*···*A*	*D*—H···*A*
C9—H9A···O2^i^	0.96	2.59	3.385 (7)	140
C9—H9C···O1^ii^	0.96	2.59	3.540 (6)	172

Symmetry codes: (i) −*x*+2, −*y*+2, −*z*+2; (ii) −*x*+2, −*y*+1, −*z*+2.
